# Seroprevalence of dengue among healthy adults in a rural community in Southern Malaysia: a pilot study

**DOI:** 10.1186/s40249-017-0384-1

**Published:** 2018-01-16

**Authors:** Amreeta Dhanoa, Sharifah Syed Hassan, Nowrozy Kamar Jahan, Daniel D. Reidpath, Quek Kia Fatt, Mohtar Pungut Ahmad, Cheong Yuet Meng, Lau Wee Ming, Anuar Zaini Zain, Maude Elvira Phipps, Iekhsan Othman, Aman Bin Rabu, Rowther Sirajudeen, Ahmad Abdul Basitz Ahmad Fatan, Faidzal Adlee Ghafar, Hamdan Bin Ahmad, Pascale Allotey

**Affiliations:** 1 Jeffrey Cheah School of Medicine and Health Sciences, Monash University Malaysia, Jalan Lagoon Selatan, Bandar Sunway, 47500 Petaling Jaya, Malaysia; 2 Segamat District Public Health Office, Ministry of Health Malaysia, Peti Surat 102, Jalan Gudang Ubat, Kampung Gubah, 85000 Segamat, Johor Darul Takzim Malaysia; 3 Infection and Immunity Cluster, Tropical Medicine and Biology Platform, Monash University Malaysia, Jalan Lagoon Selatan, Bandar Sunway, 47500 Petaling Jaya, Malaysia; 4 South East Asia Community Observatory (SEACO), 146 Jalan Sia Her Yam, Suite 601–606, Wisma Centrepoint, 85000 Segamat, Johor Darul Takzim Malaysia; 5 Hospital Segamat, Ministry of Health Malaysia, KM 6, Jalan Genuang, 85000 Segamat, Johor Darul Takzim Malaysia; 6 Universiti Sains Malaysia, 11800 Gelugor, Penang Malaysia

**Keywords:** Dengue, Dengue virus, Rural, Community-based study, Seroprevalence, Subclinical infection, Malaysia

## Abstract

**Background:**

The frequency and magnitude of dengue epidemics continue to increase exponentially in Malaysia, with a shift in the age range predominance toward adults and an expansion to rural areas. Despite this, information pertaining to the extent of transmission of dengue virus (DENV) in the rural community is lacking. This community-based pilot study was conducted to establish DENV seroprevalence amongst healthy adults in a rural district in Southern Malaysia, and to identify influencing factors.

**Methods:**

In this study undertaken between April and May 2015, a total of 277 adult participants were recruited from households across three localities in the Sungai Segamat subdistrict in Segamat district. Sera were tested for immunoglobulin G (IgG) (Panbio® Dengue Indirect IgG ELISA/high-titer capture) and immunoglobulin M (IgM) (Panbio®) antibodies. The plaque reduction neutralization test (PRNT) was conducted on random samples of IgG-positive sera for further confirmation. Medical history and a recall of previous history of dengue were collected through interviews, whereas sociodemographic information was obtained from an existing database.

**Results:**

The overall seroprevalence for DENV infection was 86.6% (240/277) (95% *CI*: 83–91%). Serological evidence of recent infection (IgM/high-titer capture IgG) was noted in 11.2% (31/277) of participants, whereas there was evidence of past infection in 75.5% (209/277) of participants (indirect IgG minus recent infections). The PRNT assay showed that the detected antibodies were indeed specific to DENV. The multivariate analysis showed that the older age group was significantly associated with past DENV infections. Seropositivity increased with age; 48.5% in the age group of <25 years to more than 85% in age group of >45 years (*P* < 0.001). No associations with occupation, study site, housing type, comorbidity, educational level, and marital status were observed, although the latter two were statistically significant in the univariate analysis. None of the studied factors were significantly associated with recent DENV infections in the multivariate analysis, although there was a pattern suggestive of recent outbreak in two study sites populated predominately by Chinese people. The majority of infections did not give rise to recognizable disease (either asymptomatic or nonspecific symptoms) as only 12.9% of participants (31/240) recalled having dengue in the past.

**Conclusions:**

The predominantly rural community under study had a very high previous exposure to dengue. The finding of a high proportion of unreported cases possibly due to subclinical infections underscores the need for enhanced surveillance and control methods. This finding also has implications for measuring disease burden, understanding transmission dynamics, and hypothesizing effects on DENV vaccine efficacy and uptake.

**Electronic supplementary material:**

The online version of this article (10.1186/s40249-017-0384-1) contains supplementary material, which is available to authorized users.

## Multilingual abstracts

Please see Additional file 1 for translations of the abstract into the five official working languages of the United Nations.

## Background

In recent decades, dengue virus (DENV) infections have become a major public health concern with an unprecedented geographic expansion worldwide. Infection with any of the four serotypes (DENV-1 – 4) belonging to the *Flavivirus* genus of the Flaviviridae family can result in clinical manifestations ranging from undifferentiated fever, classical dengue fever, to severe sometimes fatal manifestations characterized by hemorrhage and shock, known as dengue hemorrhagic fever and dengue shock syndrome, respectively [1].

It is estimated that there are 390 million DENV infections globally per year, of which 75% are subclinical [2]. Asian countries bear a disproportionately high burden of DENV infections, accounting for 70% of all cases [2]. Dengue poses a substantial economic and disease burden in Southeast Asia, with an estimated annual average of 214 000 (range: 120 000–299 000) disability-adjusted life years lost and an overall annual economic burden of US$950 million (range: US$610–1384 million) [3].

Malaysia has experienced an exponential increase in dengue cases despite having well-established dengue control measures. The dengue incidence in Malaysia increased precipitously from 31.6 cases/100 000 in 2000 to 361 cases/100 000 in 2014, i.e. an 11-fold rise [4]. Environmental changes such as rising temperatures, increased rainfall, accelerated urbanization and industrialization, population growth, and poor waste and water management, which leads to the proliferation of mosquito species (*Aedes aegypti* and *A. albopictus*) that transmit DENV, are all possible reasons for the upsurge of dengue cases [4–6].

Dengue has now become hyperendemic in many countries including Malaysia with all four DENV serotypes co-circulating, with fluctuations of the dominant serotypes over time and location [4, 5]. In recent years, there has been a shift observed in the age distribution of dengue cases toward older age groups, involving adults in the productive age group [5, 7]. Another notable shift is the rise in dengue incidence in rural areas, which is gradually approaching the high levels observed in urban communities [6, 7].

With the changing epidemiology of DENV infections in Malaysia, it has become increasingly important to obtain up-to-date seroprevalence data to further understand the distribution and burden of DENV infections. Seroprevalence studies help identify populations previously infected by dengue. This is important because the lifelong immunity developed after infection with one of the four DENV serotypes is type-specific; secondary infection by heterologous serotype is frequently, but not exclusively, associated with severe dengue manifestations [1, 2]. Seroprevalence studies reveal subclinical DENV infections, as the majority of the infections are subclinical and case reporting underestimates the true rate [2]. Patients with subclinical infections can contribute to the overall DENV transmission cycle [8]. Additionally, estimating both clinical and subclinical infections provides reliable information to support modeling for future vaccine demand and delivery strategies [2].

The aim of this pilot study was to determine the seroprevalence of DENV infections in a healthy adult population in a rural district in Southern Malaysia, and to identify factors associated with seropositivity. As a pilot study, the aim was not to establish generalizable seroprevalence rates, but to ascertain seroprevalence levels in an ostensibly healthy population.

## Methods

### Study area

The study was conducted in the catchment area of the South East Asia Community Observatory (SEACO), a Malaysian-based health and demographic surveillance site [9]. The SEACO has enumerated the population of five subdistricts in the Segamat district of Johor state, Southern Peninsular Malaysia [10]. Embedding this pilot within the SEACO facilitated the identification of a sampling frame and provided the opportunity for linkage of the data to the existing sociodemographic information in the SEACO database.

This study was restricted to Sungai Segamat, the subdistrict identified by the health department as having the highest number of reported dengue cases over the last five years. Participants were recruited from three localities (villages) within the Sungai Segamat subdistrict representing rural, urban, and semi-urban communities. Based on notified dengue cases, the three study localities had been identified as the dengue epicenter in the Sungai Segamat subdistrict since 2009 [11].

### Study population and recruitment

The comprehensive study protocol has previously been described by Jahan et al. [11], and is briefly described here. Essentially, this study extended slightly over a one-year period and consisted of four phases of data and blood collection. The first phase was undertaken between April and May 2015. Herein, we report on the first phase only, which provides information on the seroprevalence of dengue in the studied community.

As recent dengue studies in Malaysia show a shifting age distribution toward infections amongst adults [5–7], we recruited healthy adults aged ≥18 years living in the Sungai Segamat subdistrict. The exclusion criteria included: presence of fever (infrared forehead thermometer ≥37.5 °C at time of recruitment), history of yellow fever and Japanese encephalitis (JE) vaccination/disease, pregnant women, and patients with terminal illness, organ failure, mental illness, cancer, and/or underlying bleeding disorders.

The target sample was based on a sample size calculation of 333 [11]. An open invitation was sent to eligible participants through flyers dropped to their homes and was followed up with visits by SEACO data collectors to confirm participation and provide information sheets ahead of consent. Participants were then invited on a specified date to the community hall. A total of 308 agreed to participate, however, 31 did not show up. The final sample was therefore 277 (consisting of 202 Malay and 75 Chinese) participants; i.e. 83% of the target sample.

The data and blood collection was done at the local community hall. Firstly, a medical history was obtained from all participants, including a prior diagnosis of dengue, when the diagnosis was made, and whether the participant was hospitalized. All interviews were captured through a structured questionnaire using an Open Data Kit (https://opendatakit.org/) on an Android mobile device. Sociodemographic details such as age, sex, ethnicity, place of residence, marital status, highest education level, and occupation were obtained from the existing SEACO database.

Following the interview, a 5 ml blood sample was drawn from each participant by trained phlebotomists and collected in BD Vacutainer® blood collection tubes (Becton Dickinson, Franklin Lakes, NJ, USA), using a clot activator and gel separator. Proper storage and transportation of these blood samples from the community hall to the laboratory followed a standard protocol, as described elsewhere [11].

### Laboratory tests

#### DENV serology

Three serological tests were performed to detect DENV antibodies. All were enzyme-linked immunosorbent assay (ELISA) tests. First, serum samples were tested using Panbio® Dengue IgG Indirect ELISA (Alere, Brisbane, Queensland, Australia), which detects immunoglobulin G (IgG) antibodies at levels indicative of past exposure to dengue. Second, serum samples were tested using Panbio® Dengue IgG Capture ELISA (Alere, Brisbane, Queensland, Australia). This kit provides serological evidence of recent secondary DENV infections as it detects only high IgG levels (titers ≥1:2560), thereby distinguishing them from any past infections. Third, serum samples were tested using Panbio® Dengue IgM Capture ELISA (Alere, Brisbane, Queensland, Australia), with positive values indicative of recent primary DENV infections.

Serum samples were classified as negative if they tested negative on all three ELISA tests. They were classified as a recent infection if the samples tested positive for either immunoglobulin M (IgM) or high-titer IgG. They were classified as a past infection based on the Panbio® Indirect ELISA IgG, but not high-titer IgG ELISA tests [12]. All serum samples showing recent infection were tested for NS1 antigen using Panbio® Dengue Early ELISA (Alere, Brisbane, Queensland, Australia).

#### Plaque reduction neutralization test (PRNT)

An additional confirmatory test was performed using the PRNT on 96 out of 240 random samples of Panbio® Indirect ELISA IgG-seropositive serum. The PRNT is considered the gold standard for detecting serotype-specific DENV antibodies and has been widely used to measure specific DENV neutralizing antibodies, especially in studies evaluating the immunogenicity of dengue vaccines [13]. As PRNT is highly laborious, we validated the serum samples against two DENVs only i.e. DENV-1 and DENV-2, as these two were the dominant circulating DENV serotypes in Johor [14].

The PRNT was performed as previously described [15, 16], with some modifications. Virus strains used were DENV-1 (TM100; Acc: KU666942) and DENV-2 (TM213; Acc: KU666947) isolated from dengue-infected patients in Johor [14]. The PRNT was conducted in Vero cells (African green monkey kidney cells). A total of 96 patient sera together with DENV-1 and DENV-2 positive and negative sera were heat inactivated at 56 °C for 30 min and diluted at single dilution of 1:25 using an infection medium (1× MEM containing 2% inactivated FBS, 1% penicillin-streptomycin antibiotic, and 1% HEPES) as the diluent. An equal volume of diluted dengue virus (DENV-1 or DENV-2) was added to each of the patient sera and controls at the final virus concentration of 20–40 plaque-forming units (PFUs)/0.1 ml. The virus-serum mixtures were mixed well and then incubated at 37 °C for 1-h. Following this, 100 μl of the virus-serum mixture was added to the fully confluent Vero cell monolayers (in 24-well tissue culture plates) and incubated for another hour at 37 °C. Following incubation, inoculum was removed from the cells and 1 ml of 2% CMC (carboxymethyl cellulose) overlay medium was added into each well. The plates were incubated at 37 °C in the presence of 5% CO_2_ for four days. The virus controls were diluted to the final concentration of 20–40 PFUs/0.1 ml.

On day 4 post-infection, focus formation assay involving immunostaining of DENV was performed. Briefly, the overlay medium was removed and cell monolayers were fixed with cold 80% acetone for 10 min at room temperature. Cells were then incubated with a blocking buffer (wash buffer containing 1% BSA and 0.5% Triton X-100) at 37 °C in the presence of 5% CO_2_ for 45 min. Cells were incubated with primary antibody, a mouse monoclonal DENV type 1, 2, 3, and 4 antibody (GeneTex, Inc., CA, USA) at 37 °C for 1-h. A secondary antibody, alkaline phosphatase-conjugated goat anti-mouse IgG (Santa Cruz Biotechnology, Inc., CA, USA), was added onto fixed cells and the plates were incubated at 37 °C for 1-h. The foci (viral plaques) were visualized using NBT (nitrotetrazolium blue chloride) and BCIP (5-bromo-4-chloro-3′-indolyphosphate p-toluidine salt) substrate system (Bio Basic, Inc., NY, USA), and the number of plaques were counted. Serum samples were tested simultaneously against each DENV strain; each serum was tested once at one dilution of 1/50. A PRNT_70_ at 1/50 dilution cut-off was chosen to ensure that cross-reactivity with other flaviviruses could be ruled out.

The PRNT_70_ titer is defined as the reciprocal of the highest original serum dilution including the 1:2 dilution factor that was introduced by adding an equal volume of virus to the diluted serum during the neutralization step, in which viral plaque is reduced by 70% when compared to the average plaque count of virus control. In this study, a PRNT_70_ titer of ≥50 to both DENV-1 and DENV-2 or to either DENV-1 or DENV-2 alone was considered positive for DENV.

### Statistical analysis

Data were analysed using IBM SPSS Statistics for Windows, version 22.0 (IBM Corp., Armonk, NY, USA). The crude analyses were performed to determine the association of DENV seropositivity (both recent and past infection) with sociodemographic factors, comorbidities, and a past family history of dengue. Variables with a *P*-value <0.20 derived in the crude analysis were included in the final adjusted, binary logistic regression model; *P*-value <0.05 was considered statistically significant.

### Ethical approval

Ethical approval for the study was obtained from the Medical Research Ethics Committee, Ministry of Health Malaysia (NMRR-14-42-19 126), and the Monash University Human Research Ethics Committee (CF14/2543–2 014 001 379).

## Results

### General characteristics of participants

Table 1 gives the general characteristics of the 277 participants included in the study. The median age of participants was 51 years (IQR 39–61), with 65% aged ≥45 years. More than half (161, 58%) were females.

**Table 1:** Sociodemographic characteristics and seroprevalence status of participants (*N* = 277)

Characteristics	No	%	Negative	Recent infection (*N* = 31)%	Past infection (*N* = 209)%
Age group					
18–24	33	11.9	13 (39.4)	4 (12.1)	16 (48.5)
25–34	22	7.9	5 (22.7)	2 (9.1)	15 (68.2)
35–44	42	15.2	14 (33.3)	6 (14.3)	22 (52.4)
45–54	65	23.5	3 (4.6)	4 (6.2)	58 (89.2)
> 55	115	41.5	2 (1.7)	15 (13)	98 (85.2)
Gender					
Male	116	41.9	12 (10.3)	16 (13.8)	88 (75.9)
Female	161	58.1	25 (15.5)	15 (9.3)	121 (75.2)
Ethnicity					
Malay	202	72.9	33 (16.3)	17 (8.4)	152 (75.2)
Chinese	75	27.1	4 (5.3)	14 (18.7)	57 (76)
Marital status					
Divorced/widowed	25	9	1 (4)	3 (12)	21 (84)
Not married	46	16.6	15 (32.6)	6 (13)	25 (54.3)
Married	206	74.4	21 (10.2)	22 (10.7)	163 (79.1)
Highest education					
Never attended/primary	82	29.6	2 (2.4)	9 (11)	71 (86.6)
Secondary	152	54.9	23 (15.1)	17 (11.2)	112 (73.7)
College/university	43	15.5	12 (27.9)	5 (11.6)	26 (60.5)
Occupation					
Student	11	4	6 (54.5)	0	5 (45.5)
Homemaker	93	33.6	11 (11.8)	10 (10.8)	72 (77.4)
Unemployed	54	19.5	5 (9.3)	10 (18.5)	39 (72.2)
Employed	119	43	15 (12.6)	11 (9.2)	93 (78.2)
Study site					
Kampung Abdullah (urban)	60	21.7	3 (5)	11 (18.3)	46 (76.7)
Taman Segar (semi-urban)	27	9.7	3 (11.1)	4 (14.8)	20 (74.1)
Kampung Jawa (rural)	190	68.6	31 (16.3)	16 (8.4)	143 (75.3)
Type of house					
Single story terrace	190	68.6	31 (16.3)	16 (8.4)	143 (75.3)
Double story terrace	87	31.4	6 (6.9)	15 (17.2)	66 (75.9)

The participants were predominantly (68.6%) from the rural site (Kampung Jawa). However, there was smaller representation from the urban (Kampung Abdullah, 21.7%) and semi-urban (Taman Segar, 9.7%) sites as well. The majority of the participants were ethnic Malay (72.9%) and the remaining were Chinese (27.1%). The great majority of the Malays (94%) were concentrated in the rural site and the Chinese were entirely (100%) from the urban and semi-urban sites.

Forty-three percent of participants were employed and 70% had at least some secondary school education. More than one-quarter (27.1%) of the participants had at least one pre-existing comorbidity, with hypertension being the most common.

### Seroprevalence of DENV amongst participants

Serological evidence of previous exposure to DENV as denoted by a positive dengue anti-IgG indirect ELISA was identified in the serum samples of 240 (86.6%) participants (95% *CI*: 83–91%). Of the participants aged ≤24 years, 60.6% were DENV seropositive; this rose to 98.3% for participants aged ≥55 years. Of the 240 positive serum samples, 22 tested positive for high-titer IgG and another 11 were positive for IgM, indicating recent DENV infections. Two of the 33 samples were positive for both high-titer IgG and IgM. Thus, in total 11.2% (31/277) of participants (95% *CI*: 7–15%) had serological evidence of recent DENV infection and 75.5% (209/277) of participants (95% *CI*: 70–81%) had evidence of a past DENV infection. As expected, all samples tested positive for high-titer IgG were also positive for dengue anti-IgG indirect ELISA. None of the serum samples with recent DENV infections tested positive for NS1 antigen.

**Fig. 1:**
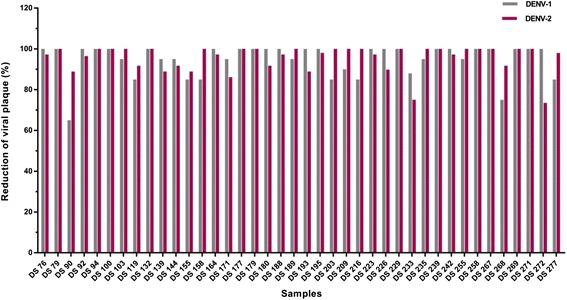
The PRNT conducted on 40 representative patient sera against DENV-1 and DENV-2. Patient sera were diluted in 1:25 and incubated with 20–40 PFUs of DENV-1 or DENV-2 for 1-h at 37 °C. Each virus-serum mixture was then inoculated onto Vero cell monolayer and incubated at 37 °C in the presence of 5% CO_2_ for 1-h. After virus adsorption, inoculums were removed from the cells, which was followed by the addition of CMC overlay medium. Plates were incubated at 37 °C under 5% CO_2_ for four days. The viral plaques were then detected and visualized via focus formation assay. The percentage of plaque reduction was calculated relative to the number of plaques in the virus controls

**Fig. 2:**
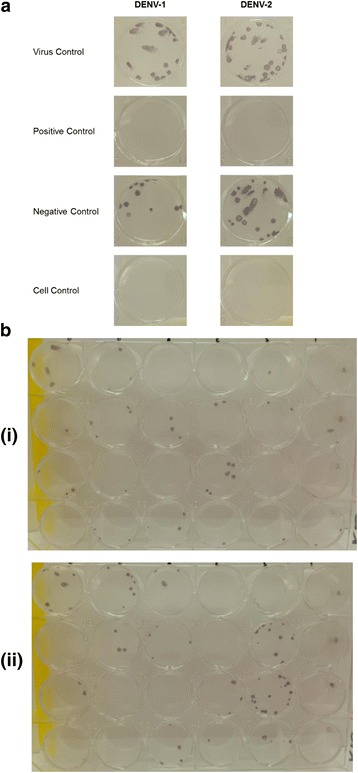
Plaque formations of DENV-1 and DENV-2 of a focus forming assay, PRNT in 24-well plates. **a** Morphology of DENV-1 and DENV-2 in virus control and negative serum control wells of a-24 well plate (magnified 2 ×). Patient sera with PRNT_70_ titer of >625 against both DENV-1 and DENV-2 were used as the positive serum controls, whilst healthy human serum was used as a negative control. **b** A proportion of the 24 tested patient sera against DENV-1 (i) and DENV-2 (ii). Patient sera (diluted 1:25) were incubated with 20–40 PFUs of DENV-1 or DENV-2 for 1-h at 37 °C, followed by adsorption for 1-h on Vero cells monolayer, overlaid with CMC medium and further incubated for four days. The infected cells or plaques were immunostained with a mouse monoclonal dengue virus type 1, 2, 3, and 4 antibody [D1–11(3)] (GeneTex, Inc.) followed by alkaline phosphatase-conjugated anti-mouse IgG, and viral plaques were visualised using NBT-BCIP substrate

All 96 randomly selected samples showed at least 50% plaque reduction for both DENV-1 and DENV-2 or either DENV-1 or DENV-2. The PRNT conducted on 40 of these samples is shown in Fig. 1. However, to further minimize cross-reactivity with other flaviviruses, a more stringent value of at least 70% plaque reduction (PRNT_70_) for both DENV-1 and DENV-2 or either DENV-1 or DENV-2 was considered positive or specific to DENVs. All the tested sera samples showed a PRNT_70_ titer ≥50, indicating they were indeed specific to DENV antibodies. Figure 2a and 2b are representative images of PRNT showing neutralisation along with the positive and negative controls.

### Past DENV infections

The univariate analysis showing the comparison between participants with and without past DENV infections is summarized in Table 2.

**Table 2:** Factors associated with past DENV infections (*N* = 209)

				Univariate analysis			Multivariate Analysis		
Variable	Total participants ^a^	No positive/No tested	%	Crude *OR*	95% *CI*	*P* value	Adjusted *OR*	95% *CI*	*P* value
Age group						< 0.001			0.001
18–24	33	16/33	48.5	1.0	Referent		1.0	Referent	–
25–34	22	15/22	68.2	2.28	0.74–7.03	0.153	1.679	0.353–7.984	0.515
35–44	42	22/42	52.4	1.17	0.47–2.91	0.738	0.780	0.172–3.541	0.748
45–54	65	58/65	89.2	8.80	3.11–24.9	< 0.001	5.938	1.110–31.771	0.037
55 and above	115	98/115	85.2	6.12	2.60–14.40	< 0.001	4.165	0.830–20.894	0.083
Gender						0.893			
Male	116	88/116	75.9	1.04	0.60–1.81	0.893			
Female	161	121/161	75.2	1.00	Referent				
Ethnicity						0.897			
Malay	202	152/202	75.2	1.00	Referent				
Chinese	75	57/75	76	1.04	0.56–1.93	0.897			
Marital status						0.002			0.963
Divorced/separated	25	21/25	84	1.39	0.45–4.25	0.569	0.969	0.286–3.282	0.959
Not married	46	25/46	54.3	0.31	0.16–0.61	0.001	0.839	0.236–2.982	0.787
Married	206	163/206	79.1	1.00	Referent		1.0	Referent	–
Highest education						0.006			0.485
Never attended/Primary	82	71/82	86.6	4.22	1.75–10.20	0.001	2.032	0.637–6.478	0.231
Secondary	152	112/152	73.7	1.83	0.90–3.72	0.095	1.394	0.573–3.395	0.464
College/university	43	26/43	60.5	1.0	Referent		1.0	Referent	–
Occupation						0.132			0.548
Student	11	5/11	45.5	1.0	Referent		1.0	Referent	–
Homemaker	93	72/93	77.4	4.11	1.14–14.83	0.031	0.813	0.145–4.570	0.815
Unemployed	54	39/54	72.2	3.12	0.83–11.77	0.093	0.796	0.161–3.928	0.780
Employed	119	93/119	78.2	4.29	1.21–15.19	0.024	1.326	0.266–6.624	0.731
Study site						0.961			
Kampung Jawa (rural)	190	143/190	75.3	1.0	Referent				
Kampung Abdullah (urban)	60	46/60	76.7	1.08	0.54–2.14	0.825			
Taman Segar (semi-urban)	27	20/27	74.1	0.94	0.37–2.36	0.894			
Type of housing						0.914			
Single terrace	190	143/190	75.3	1.0	Referent				
Double terrace	87	66/87	75.9	1.03	0.57–1.87	0.914			
Comorbidity						0.168			0.546
No	202	148/202	73.3	1.0	Referent		1.0	Referent	–
Yes	75	61/75	81.3	1.59	0.82–3.07	0.168	0.767	0.325–1.812	0.546
No of medical illness						0.387			
0	202	148/202	73.3	1.0	Referent				
1	48	39/48	81.2	1.58	0.72–3.48	0.255			
> 1	27	22/27	81.5	1.60	0.58–4.45	0.363			
Hypertension						0.325			
No	225	167/225	74.2	1.0	Referent				
Yes	52	42/52	80.8	1.46	0.69–3.09	0.325			
Diabetes						0.163			0.449
No	253	188/253	74.3	1.0	Referent		1.0	Referent	–
Yes	24	21/24	87.5	2.420	0.70–8.38	0.163	1.735	0.416–7.224	0.449
High cholesterol						0.498			
No	260	195/260	75	1.0	Referent				
Yes	17	14/17	82.4	1.56	0.43–5.58	0.498			
Asthma						0.803			
No	270	204/207	75.6	1.24	0.23–6.52	0.803			
Yes	7	5/7	71.4	1.0	Referent				
Family history of dengue						0.269			
No	217	167/217	77	1.43	0.76–2.70	0.269			
Yes	60	42/60	70	1.0	Referent				
Family members with dengue						0.969			
1	49	34/49	69.4	1.0	Referent				
> 1	10	7/10	70	1.029	0.23–4.53	0.969			

^a^Total number of participants is the denominator for each variable category

Seropositivity generally increased with age, rising from 48.5% in the age group <25 years to more than 85% in age group of >45 years (*P* < 0.001). The highest prevalence (89.2%) was noted amongst participants in the age group of 45–54 years. Non-married participants had significantly lower seropositivity rates (54.3%) compared to those who were married or divorced/separated. Seropositivity was inversely related to the level of education. It was highest amongst participants who never attended school or had a primary level education (86.6%) and lowest amongst those who attended college or university (60.5%) (*P* = 0.006). No significant differences were detected between seropositivity and sex, ethnicity, occupation, or comorbidity. Students had lower seroprevalence (45.5%) compared to the employed, unemployed, and housewives who had higher rates in the range of 72–78%; however, the differences were not statistically significant. There was no difference in seropositivity amongst participants residing in the urban (76.7%), semi-urban (74.1%), or rural sites (75.3%) or between the types of houses they resided in (single-story verses double-story terrace). Similarly, history of family members with dengue did not determine seropositivity, with only 20.1% (42/209) of participants with past dengue infections reporting a family history of dengue.

In the multivariate logistic regression model, only age group remained significantly associated with the risk of past DENV infections. All other variables were not significant (see Table 2).

### Recent DENV infections

The univariate analysis showing the comparison between participants with and without recent DENV infections is summarized in Table 3.

**Table 3:** Factors associated with recent DENV infections (*N* = 31)

				Univariate Analysis			Multivariate Analysis		
Variable	All participants ^a^	No positive/No tested	%	Crude *OR*	95% *CI*	*P value*	Adjusted *OR*	95% *CI*	*P value*
Age group						0.649			
18–24	33	4/33	12.1	1.0	Referent				
25–34	22	2/22	9.1	0.72	0.12–4.34	0.72			
35–44	42	6/42	14.3	1.21	0.31–4.69	0.785			
45–54	65	4/65	6.2	0.47	0.11–2.04	0.32			
55 and above	115	15/115	13	1.09	0.33–3.53	0.889			
Gender						0.246			
Male	116	16/116	13.8	1.56	0.74–3.29	0.246			
Female	161	15/161	9.3	1.0	Referent				
Ethnicity						0.019			0.467
Malay	202	17/202	8.4	1.0	Referent		1.0	Referent	–
Chinese	75	14/75	18.7	2.50	1.16–5.36	0.019	2.490	0.213–29.097	0.467
Marital status						0.892			
Divorced/separated	25	3/25	12	1.14	0.32–4.12	0.841			
Not married	46	6/46	13	1.25	0.48–3.29	0.645			
Married	206	22/206	10.7	1.0	Referent				
Highest education						0.994			
Never attended/Primary	82	9/82	11	0.937	0.293–2.993	0.913			
Secondary	152	17/152	11.2	0.957	0.332–2.763	0.935			
College/university	43	5/43	11.6	1.0	Referent				
Occupation						0.174			0.245
Student/Homemaker	104	10/104	9.6	1.04	0.42–2.57	0.924	0.969	0.386–2.433	0.947
Unemployed	54	10/54	18.5	2.23	0.89–5.63	0.089	2.064	0.785–5.425	0.142
Employed	119	11/119	9.2	1.0	Referent		1.0	Referent	–
Housing						0.034			0.839
Single story terrace	190	16/190	8.4	1.0	Referent		1.0	Referent	–
Double story terrace	87	15/87	17.2	2.27	1.06–4.82	0.034	0.8	0.092–6.928	0.839
Study site						0.095			0.886
Kampung Jawa (rural)	190	16/190	8.4	1.0	Referent		1.0	Referent	–
Kampung Abdullah(urban)	60	11/60	18.3	2.44	1.06–5.60	0.035	0.897	0.205–3.926	0.886
Taman Segar(semi-urban)	27	4/27	14.8	1.89	0.58–6.15	0.289			
Comorbidity						0.126			0.933
No	202	19/202	9.4	1.0	Referent		1.0	Referent	–
Yes	75	12/75	16	1.83	0.84–3.99	0.126	0.942	0.233–3.806	0.933
No of medical illness						0.302			
0	202	19/202	9.4	1.0	Referent				
1	48	8/48	16.7	1.93	0.79–4.71	0.151			
> 1	27	4/27	14.8	1.67	0.52–5.35	0.384			
Hypertension						0.126			0.432
No	225	22/225	9.8	1.0	Referent		1.0	Referent	–
Yes	52	9/52	17.3	1.93	0.83–4.48	0.126	1.816	0.410–8.045	0.432
Diabetes						0.832			
No	253	28/253	11.1	1.0	Referent				
Yes	24	3/24	12.5	1.15	0.32–4.09	0.832			
High cholesterol						0.389			
No	260	28/260	10.8	1.0	Referent				
Yes	17	3/17	17.6	1.78	0.48–6.56	0.389			
Asthma						0.793			
No	270	30/270	11.1	1.0	Referent				
Yes	7	1/7	14.3	1.33	0.15–11.45	0.793			
Past history of dengue (personal)						0.000			
No	246	20/246	8.1	1.0	Referent				
Yes	31	11/31	35.5	6.21	2.61–14.78	0.000			
Family history of dengue						0.017			0.082
No	217	19/217	8.8	1.0	Referent		1.0	Referent	–
Yes	60	12/60	20	2.60	1.18–5.73	0.017	2.186	0.906–5.274	0.082
Family members with dengue infections						0.977			
1	49	10/49	20.4	1.03	0.18–5.60	0.977			
> 1	10	2/10	20	1.0	Referent				

^a^Total number of participants is the denominator for each variable category

Seroprevalence was significantly higher in the Chinese ethnic group (*P* = 0.019). Although the three study sites had similar seroprevalence rates for past DENV infections, participants living in the urban and semi-urban sites, where the Chinese predominate, had higher serological evidence of recent infection compared to the rural site, which is populated predominantly by Malays (*P* = 0.095). Participants living in double-story terrace houses had significantly higher rates of recent DENV infections compared to those living in single-story terrace houses (*P* = 0.034). There was a significantly higher prevalence of recent DENV infection in participants who reported having family members with a diagnosis of dengue fever (*P* = 0.017); with 38.7% (12/31) reporting a family history. Contrary to past infections, there was no difference in seroprevalence amongst participants with recent infections in the various age groups. Similarly, sex, marital status, occupation, education level, and comorbidity were not risk factors for recent DENV infections.

In the multivariate logistic regression model, none of these variables independently predicted recent infections. However, a family history of dengue almost reached statistical significance (*P* = 0.082) and showed a higher risk of recent infections (a*OR*: 2.186, 95% *CI* = 0.906–5.274).

### Recall of history of dengue

Overall, only 12.9% (31/240) of participants recalled having had dengue, despite very high serological evidence of previous DENV exposure. All participants who recalled having dengue in the past showed dengue IgG seropositivity. Of these, 18 were hospitalized and another 13 were treated on an outpatient basis, and all had complete resolution of symptoms. Amongst participants who had serological evidence suggesting past DENV infections, only 9.6% (20/209) reported having dengue previously. Amongst those with recent DENV infections, 35.5% (11/31) recalled having dengue.

## Discussion

To the best of our knowledge, this is the first population-based household survey assessing DENV seroprevalence amongst healthy Malaysian adults. The results of this study provide evidence that there was very high prevalence of exposure to DENV in the studied rural community in Southern Malaysia, in an ostensibly healthy population.

The high seroprevalence found in this study mirrors another nationwide cross-sectional study conducted in Malaysia, in which 91.6% of healthy adults aged 35–74 years were DENV seropositive [6]. However, studies conducted amongst the urban population attending primary health clinics in Malaysia reported lower rates, ranging between 61% and 67% [7]. While the seroprevalence rates in our study were higher compared to those amongst adults in Singapore [12, 17], Pakistan [18], Venezuela [19], and Saudi Arabia [20], countries such as American Samoa [21] and South India [22] have reported higher rates, with almost universal exposure to DENV.

With 192 reported clinical dengue cases in Segamat in 2014 [11], the incidence of dengue in the district is generally lower (104.72/100 000 population) compared to a national incidence of 361 cases/100 000 for the same year [4]. In fact, there were only five reported cases of dengue from the three study sites in 2014 based on the official system of notification obtained from the Segamat District Public Health Office database: urban (*n* = 3), rural (*n* = 0), and semi-urban (*n* = 2) sites.

Despite having relatively low incidence of reported dengue cases in Segamat district, an overwhelming 86.6% of participants had serological evidence of previous DENV exposure, out of which 11.2% were recent exposures. This discrepancy could be attributed to the dengue notification system in Malaysia, which is based on passive reporting and may not reflect the actual disease burden due to underreporting, misreporting [5, 22], and the failure to capture subclinical infections [2]. This disparity is further supported by the finding that, overall, only 12.9% of participants with serological evidence of dengue exposure had recollection of having had a dengue infection. They were not diagnosed clinically by a healthcare professional, as they were either asymptomatic or had minimal symptoms not necessitating medical attention or hospitalization. The nonspecific nature of symptoms misattributing it to other febrile illnesses may partly account for this [22]. Our findings suggest a possibility that a high number of subclinical dengue cases are present in this community. This corroborates the iceberg phenomena that most dengue infections are clinically unapparent and are consistent with high rates reported in countries such as Singapore [17], South India [22], and Saudi Arabia [20].

The multivariate analysis showed that the older age was the only factor associated with past DENV infections. The prevalence of IgG antibodies increased with increasing age, with >98% of the population having been exposed to dengue after the age of 55 years. Several studies have shown an association between age and IgG dengue seropositivity [6, 7, 12, 17, 20, 22], consistent with the long-term persistence of anti-DENV IgG once a person is infected [1]. The increased seroprevalence with age suggests that the longer a person resides in an endemic area, the higher the chance of being infected by DENV [19, 20]. Ethnicity and being of the male sex, which have previously been reported as risk factors for past DENV infections [12, 18, 20], were not found to be associated in our analysis and other studies [6, 17]. Concurring with several studies [12, 18], we found no statistical association between past DENV infections and occupation. Although lower educational attainment was associated with higher seroprevalence, it did not remain a significant risk factor after controlling for other risk factors. Previous studies have shown a differing relationship when assessing the impact of income, education, structural housing condition, overcrowding, and socioeconomic status on DENV infections [23]. Although some studies have shown an inverse association between DENV infections and socioeconomic status [19, 22], other studies showed no clear association [23]. The type of housing, which was either single-story or double-story terrace, did not determine seropositivity. In a Malaysian study, the incidence of dengue was higher in areas with interconnected houses (terrace houses, apartments, and flats) as compared to independent houses [24], although such a comparison was not possible in our study as all participants lived in terrace houses.

Studies have shown an association between water storage in containers [19] and past dengue infections. Although this factor was not measured in our study, all households in our study sites have availability of pipe water and sewage system, reducing the need to store water in containers, which are potential breeding grounds for mosquitoes. A notable finding was that 75.3% of participants in the rural site had been exposed to dengue, with no difference between the rural, urban, or semi-urban sites. Although previously regarded a disease of urban areas in Malaysia [5], recent studies suggest its extent to rural population as well [6, 7]. Population mixing facilitated by the efficiency of modern transportation networks, growing vector population, and changes in the usage of agricultural land in rural areas have contributed to homogeneousness in dengue risk between urban and rural areas [6, 7].

Serological evidence of recent infection was noted in 11.2% of participants. These rates were comparable to those in Venezuela [19], whereas higher rates were reported in South India [22]. Conversely a study conducted in Singapore [12] reported only 2.6% of recent infections. Contrary to the association of age with past DENV infection, recent infection did not display age differences. Chinese ethnicity, housing type, study site, and family history were associated with recent dengue infection in the univariate analysis. It seems likely that this pattern resulted from recent outbreaks with silent transmission of DENV in urban and semi-urban study sites that are populated predominantly by the Chinese. However, none of these factors remained significant predictors of recent infection in the multivariate analysis.

### Limitations

The key limitation we faced in this study was the relatively small sample size from a single study site (with three sub-sites). However, this pilot study was conducted to ascertain DENV seroprevalence in an ostensible population, rather than establishing generalizable seroprevalence rates. Furthermore, despite the relatively small number, there was a very high exposure of the studied population to DENV, providing evidence for the need to perform larger-scale prospective longitudinal studies, and perhaps even revising the current surveillance system.

A limitation of dengue serological surveys is the known cross-reactivity with other flaviviruses such as JE and yellow fever. However, the high IgG seropositivity in our study most likely reflects the actual prevalence of DENV in the studied community, as there have been no reported cases of JE in Segamat district. Furthermore, other flaviviruses such as yellow fever and West Nile virus have never been reported or recognized in Malaysia, and each of the participants were explicitly asked whether they had been vaccinated for yellow fever or JE virus. In addition, this study was conducted prior to the introduction of Zika virus and there have been no reported cases of Zika virus infections in the Segamat district. Most convincingly, the PRNT assay performed on random samples of IgG-positive sera showed that the detected antibodies were indeed specific to DENV.

Certain amount of recall bias is expected when interviewing people about past diagnosis of dengue. However, this is likely to be minimal because in a dengue endemic country, vigilance and awareness about dengue is expected to be higher.

## Conclusions

Data from this pilot study provide preliminary evidence that people of the Segamat rural community in Southern Malaysia had a very high previous exposure to DENV. Despite this, below 13% of participants recalled having dengue in the past, suggesting a potentially large reservoir of subclinical infection with unrecognized transmission lurking in this community, undetected by the official surveillance system. Future larger-scale studies should be conducted, as the findings have implications for enumerating disease burden, hypothesizing effects on DENV vaccine efficacy and uptake, determining disease severity and transmission dynamics, and identifying high-risk areas to target dengue prevention and control activities [2, 25]. This study also provides valuable information that can inform public information campaigns about exposure to dengue.

## Additional file

Additional file 1: Multilingual abstracts in the five official working languages of the United Nations. (PDF 552 kb)

## Abbreviations

a*OR*: Adjusted odds ratio
*CI*: Confidence interval
DENV: Dengue virus
ELISA: Enzyme-linked immunosorbent assay
IgG: Immunoglobulin G
IgM: Immunoglobulin M
JE: Japanese encephalitis
*OR*: Odds ratio
PFU: Plaque-forming unit
PRNT: Plaque reduction neutralization test
SEACO: South East Asia Community Observatory
