# Supplementary material for: Seroprevalence of dengue among healthy adults in a rural community in Southern Malaysia: a pilot study
Source: Infect Dis Poverty. 2018 Jan 16;7:1. doi: 10.1186/s40249-017-0384-1 (PMC5769361; doi:10.1186/s40249-017-0384-1)
Supplement: Additional file 1: — Multilingual abstracts in the five official working languages of the United Nations. (PDF 552 kb) [file 40249_2017_384_MOESM1_ESM.pdf]

Translation of the abstract into the five official working languages of the United Nations

الانتشار المصلي لحُمى الضنك بين البالغين الأصحاء في مجتمع ريفي بجنوب ماليزيا: دراسة تمهيدية

عامريتا ظانوا، شريفة سيد حسن، نوروزي قمر جهان، دانيال د ريدباث، كيك كيا فات، مهتار بنجت أحمد، شيونج يوت مينج، لوا وي مينج، دكتور أنور زيني زين، دكتور ماودي إلفيرا فيبس، دكتور جيخسان عثمان، عمان بن ربه، روثر سراج الدين، أحمد عبد الباسط أحمد فتان، دكتور فيصل عدلي غفار، دكتور حمدان بن أحمد، وبسكال اللوطي

الملخص

الخلفية: تستمر وتيرة وباء حمى الضنك وحجمه في الزيادة أضعاف مضاعفة في ماليزيا، مع تحول في الفئة العمرية الغالبة إلى البالغين، وتوسع في المناطق الريفية. وبرغم هذا، يوجد فقر في المعلومات المتعلقة بمدى انتقال فيروس حمى الضنك في المجتمع الريفي. أجريت هذه الدراسة التمهيدية المبنية على المجتمع لتحديد الانتشار المصلي لفيروس حمى الضنك بين البالغين الأصحاء في منطقة ريفية بجنوب ماليزيا، وللتعرف على العوامل المؤثرة.

المنهجيات: في هذه الدراسة التي أجريت بين إبريل ومايو ٢٠١٥، تم تعيين ٢٧٧ مشاركًا من البالغين من أسر عبر ثلاث محليات في المقاطعة الفرعية سنجاي سيجامات، في مقاطعة سيجامات. وتم فحص الأمصال للأجسام المضادة للجلوبولين المناعي ج (IgG) (التقاط مقايسة الممتز المناعي المرتبط بالإنزيم ELISA أو أعلى معيار للجلوبولين المناعي ج غير المباشر لحمى الضنك لبانبيو) والجلوبولين المناعي م (IgM) (بانبيو). وأجري اختبار تحييد الانخفاض اللويحي (PRNT) على عينات عشوائية لمصل الجلوبولين المناعي ج الإيجابي لمزيد من التأكيد. وتم جمع السجل الطبي واسترجاع سجل سابق لحمى الضنك عبر مقابلات، في حين تم الحصول على معلومات ديمجرافية اجتماعية من قاعدة بيانات حالية.

النتائج: وصلت نسبة الانتشار المصلي لعدوى فيروس حمى الضنك إلى ٨٦،٦٪ (٢٧٠\٢٤٠) (٩٥٪ مجال الثقة: ٨٣٪-٩١٪). كما لوحظت الأدلة المصلية لعدوى حديثة (الجلوبولين المناعي م\التقاط أعلى معيار للجلوبولين المناعي ج) في ١١،٢٪ (٢٧٧\٣١) من المشاركين، في حين ظهرت أدلة على عدوى سابقة في ٧٥،٤٪ (٢٧٧\٢٠٩) من المشاركين (الجلوبولين المناعي ج غير المباشر بدون العدوى الحديثة). وأظهر فحص اختبار تحييد الانخفاض اللويحي (PRNT) أن الأجسام المضادة التي اكتشفت هي بالفعل محددة لفيروس حمى الضنك. كما أظهر التحليل متعدد المتغيرات الارتباط الشديد للفئة العمرية الأكبر سنًا بعدوى حمى الضنك سابقًا. وارتفعت الإيجابية المصلية مع العمر: ٤٨،٥٪ للفئة العمرية <٢٥ عامًا، لتصل لما يزيد على ٨٥٪ للفئة العمرية >٤٥ (P< ٠،٠٠١). ولم تُلحظ أي صلة بين المهنة، وموقع الدراسة، ونوع السكن، والاعتلال المشترك، ومستوى التعليم، والحالة الاجتماعية، رغم أن آخر اثنين كانا ذو دلالة إحصائية في التحليل أحادي المتغير. ولم تكن لأي من العوامل المدروسة صلة كبيرة بعدوى فيروس حمى الضنك الحديثة في التحليل أحادي المتغير، ولكن وُجد نمط يوحي باندلاع حديث في موقعين من مواقع الدراسة التي يسكنها في الغالب الشعب الصيني. ولم تتسبب العدوى بالأغلبية في أمراض يمكن التعرف عليها (إما عديمة الأعراض أو أعراض غير محددة)، حيث تذكر ١٢،٩٪ فقط من المشاركين (٢٤٠\٣١) تعرضهم لحمى الضنك في السابق.

الخاتمة: تعرض المجتمع الذي أجري عليه الدراسة والذي يغلب عليه الطابع الريفي إلى حمى الضنك بشكل كبير في السابق. وتؤكد نتيجة النسبة العالية للحالات غير المبلغ عنها ربما بسبب العدوى عديمة الأعراض على الحاجة إلى تحسين منهجيات المراقبة والسيطرة. كما أن لهذه النتيجة أيضًا آثار على قياس حموية المرض، وفهم ديناميات انتقال العدوى، وافتراض آثار فعالية مصل فيروس حمى الضنك وامتصاصه.

Translated from English version into Arabic by Nema Alaraby, through

TRANSLATORS
WITHOUT BORDERS

马来西亚南部农村社区健康成人登革热血清阳性率：一项试点研究

Amreeta Dhanoa, Sharifah Syed Hassan, Nowrozy Kamar Jahan, Daniel D Reidpath, Quek Kia Fatt, Mohtar Pungut Ahmad, Cheong Yuet Meng, Lau Wee Ming, Prof. Anuar Zaini Zain, Prof. Maude Elvira Phipps, Prof. Iekhsan Othman, Aman Bin Rabu, Rowther Sirajudeen, Ahmad AbdulBasitz Ahmad Fatan, Dr. Faidzal Adlee Ghafar, Dr. Hamdan Bin Ahmad and Pascale Allotey

摘要

引言：马来西亚登革热疫情的频率和规模呈指数级持续增长，流行的年龄范围逐渐转向成年人，并不断扩张到农村地区。然而，目前仍然缺乏登革热病毒(DENV)在农村地区传播程度的相关信息。作者在马来西亚南部的一个农村地区开展了以社区为基础的试点研究，以确定健康成年人的DENV血清流行率，并确定影响因素。

方法：2015 年 4 月至 5 月，本研究共纳入 277 名成年参与者，分别来自 Segamat 地区 Sungai Segamat 亚区的三个地区的家庭。通过血清检测免疫球蛋白 G(免疫球蛋白)(Panbio®登革热间接免疫球蛋白 ELISA / high-titer capture)和免疫球蛋白 M(IgM)(Panbio®)抗体。对 IgG-阳性血清随机样本进行血小板减少中和试验以进一步确认结果。通过访谈收集病史，回顾了以往的登革热病史，并从现有的数据库中获取社会人口信息。

结果：总体 DENV 血清阳性率为 86.6%(240/277)(95% *CI*:83–91%)。在 11.2%(31 / 277)的参与者中发现新近感染(IgM /high-titer capture IgG)的血清学证据，而在 75.4%(209 / 277)的参与者中存在既往感染的证据(间接 IgG 减去新近感染)。PRNT 检测显示检测到的抗体的确呈 DENV 特异性。多变量分析显示，年纪较大的人群与既往 DENV 感染显著相关。血清阳性随着年龄的增加而增加。在小于 25 岁年龄组为 48.5%，在大于 45 岁年龄组中为 85%以上($P < 0.001$)。单变量分析没有发现其与职业、研究地点、住房类型、共患病、教育水平和婚姻状况的关联性，尽管后两者在单变量分析中具有统计学意义。在多变量分析中，没有研究因素与新近DENV感染有显著的相关性，尽管最近暴发的两个地点在中国人较多的居住地。大多数感染没有导致可识别的疾病(无症状或非特异性症状)，只有 12.9%的参与者(31/240)能够回忆起在过去曾感染过登革热。

结论：在本研究中，主要农村社区对此前登革热的暴露程度很高。研究发现未报告病例的比例较高，可能是由亚临床感染所导致的，这表明需要加强监测和控制方法。这一发现也对确定疾病负担、了解传播动力学、以及对 DENV 疫苗功效的预测有重要的意义。

Translated from English version into Chinese by Xin-Yu Feng, through

CHINESE CENTER FOR DISEASE CONTROL AND PREVENTION
NATIONAL INSTITUTE OF PARASITIC DISEASES

Séroprévalence de la dengue auprès d'adultes sains dans une communauté rurale du sud de la Malaisie : une étude pilote

Amreeta Dhanoa, Sharifah Syed Hassan, Nowrozy Kamar Jahan, Daniel D Reidpath, Quek Kia Fatt, Mohtar Pungut Ahmad, Cheong Yuet Meng, Lau Wee Ming, prof. Anuar Zaini Zain, prof. Maude Elvira Phipps, prof. Iekhsan Othman, Aman Bin Rabu, Rowther Sirajudeen, Ahmad AbdulBasitz, Ahmad Fatan, dr Faidzal Adlee Ghafar, dr Hamdan Bin Ahmad et Pascale Allotey

RÉSUMÉ

Contexte : La fréquence et l'ampleur des épidémies de dengue ne cessent d'augmenter de façon exponentielle en Malaisie; de plus, ces épidémies frappent désormais davantage les adultes et les régions à prédominance rurale. Malgré cela, les données relatives à l'étendue de la transmission du virus de la dengue (DENV) dans la communauté rurale font défaut. Cet essai pilote communautaire a été mené auprès d'adultes sains dans une communauté rurale du sud de la Malaisie pour déterminer la séroprévalence du DENV et pour déterminer les facteurs déterminants.

Méthodes : Dans cette étude menée entre avril et mai 2015, un total de 277 participants adultes ont été recrutés dans trois localités du sous-district de Sungai Segamat (district de Segamat). Les sérums ont été testés pour détecter les anticorps spécifiques à l'immunoglobuline G (IgG) (Panbio® Dengue IgG indirect Capture Elisa) et à l'immunoglobuline M (IgM) (Panbio®). La technique de réduction de plages de lyse causées par le virus (*plaque reduction neutralization tes*t [PRNT]) a été utilisée sur des échantillons aléatoires de sérums positifs en IgG en vue d'une confirmation ultérieure. Les antécédents médicaux et le rappel des antécédents de dengue ont été recueillis au moyen d'entrevues. Les renseignements sociodémographiques ont été obtenus à partir d'une base de données existante.

Résultats : La séroprévalence globale de l'infection au DENV était de 86,6 % (240/277) (IC de 95 % : 83–91 %). Les tests sérologiques confirment la présence d'infection récente (IgM/capture IgG à un haut élevé) chez 11,2 % (31/277) des participants et la présence de signes d'infection antérieure chez 75,4 % (209/277) des participants (IgG indirect moins les infections récentes). Le test PRNT a montré que les anticorps détectés étaient bien spécifiques au DENV. L'analyse multivariée a montré une association significative entre le groupe de sujets le plus âgé et les infections antérieures au DENV. La séropositivité augmentait avec l'âge : 48,5 % chez les adultes de moins de 25 ans et plus de 85 % chez ceux de plus de 45 ans (p < 0,001). Aucune association n'a été observée avec la profession, le lieu d'étude, le type de logement, la comorbidité, le niveau de scolarité et l'état matrimonial, bien que ces deux derniers facteurs aient été statistiquement significatifs dans l'analyse univariée. Aucun des facteurs étudiés dans l'analyse multivariée n'a été associé de façon significative à des infections récentes au DENV. Toutefois, il semble y avoir eu une éclosion récente dans deux sites d'étude peuplés principalement par des Chinois. La majorité des infections n'ont pas donné lieu à une maladie reconnaissable (asymptomatiques ou symptômes non spécifiques) puisque seulement 12,9 % des participants (31/240) se rappelaient avoir eu la dengue dans le passé.

Conclusion : La communauté à l'étude, principalement rurale, a déjà été très exposée à la dengue. La constatation d'une forte proportion de cas non déclarés, peut-être en raison d'infections subcliniques, souligne la nécessité d'améliorer les méthodes de surveillance et de contrôle. Ce résultat a également des répercussions sur la mesure du fardeau de la maladie, la compréhension de la dynamique de la transmission et les effets hypothétiques sur l'efficacité et l'adoption du vaccin DENV.

Translated from English version into French by Lina Scarpellini, through

TRANSLATORS
WITHOUT BORDERS

Доминирование серотипа лихорадки Денге среди здорового взрослого населения сельского сообщества Южной Малазии: Экспериментальное исследование

Амрита Дханоа, Шарифа Сиед Хассан, Новрози Камар Джахан, Даниел Д Райдпат, Квет Киа Фатт, Мохтар Пунгут Ахмад, Чэон Юэт Мен, Лау Вээ Мин, проф. Ануар Заини Заин, проф. Мод Эльвира Фиппс, проф. Иекхсан Осман, Амам Бин Рабу, Раузер Сираджудин, Ахмад АбдулБасиц Ахмад Фатан, доктор Файдзал Адли Гхафар, доктор Хамдан Бин Ахмад и Паскаль Аллотей

АННОТАЦИЯ

Справочная информация: Частота и масштаб эпидемий лихорадки Денге в Малазии возрастают в геометрической прогрессии, при этом наблюдается смещение возрастной категории преимущественно в сторону взрослого населения, а также расширение охвата в сельских районах. Несмотря на это, отсутствует информация о степени заражаемости *вирусом Денге* (DENV) в сельских сообществах. Целью данного экспериментального исследования, проведённого на уровне общин, являлось установить доминирование серотипа DENV среди здорового взрослого населения сельского региона в Северной Малазии, а также выявить факторы влияния.

Методы: В проведённом с апреля по май 2015 года исследовании были задействованы в общей сложности 277 взрослых участников из домохозяйств трёх населённых пунктов подрайона Сунгай Сегамат, находящихся в административном районе Сегамат. Был произведён анализ сыворотки на присутствие антител иммуноглобулина группы G (IgG) (Panbio® Dengue Indirect IgG ELISA/насыщенное извлечение) и иммуноглобулина группы M (IgM) (Panbio®). Для дальнейшего подтверждения на случайной выборке сывороток IgG(+) была выполнена реакция нейтрализации бляшкообразования (PRNT). Сбор информации относительно медицинских историй болезни, а также предыдущих случаев заболевания лихорадкой Денге производился посредством собеседований, тогда как социо-демографические данные были получены из существующей базы данных.

Результаты: Общее доминирование серотипа заражаемости по DENV составило 86,6% (240/277) (95% CI: 83–91%). Присутствие серологических данных о недавних инфецированиях (IgM/насыщенное извлечение IgG) было замечено у 11,2% (31/277) участников, тогда как признаки прошлых инфецирований наблюдались у 75,4% (209/277) участников (непрямое IgG за минусом недавних инфецирований). Анализ реакции нейтрализации бляшкообразования продемонстрировал, что выявленные антитела действительно оказались характерными к вирусу Денге. Многофактроный анализ показал, что прошлые инфецирования вирусом Денге значительно чаще наблюдались в старших возрастных группах. Серопозитивная реакция увеличивалась с возрастом: от 48,5% в возрастной группе <25 лет до более чем 85% в возрастной группе >45 лет ($P < 0,001$). Несмотря отсутствие выявления зависимости показателей от профессии, места проведения исследования, условий проживания, коморбидности, уровня образования или семейного положения, последние две составляющие были расценены в качестве статистически значимых элементов при однофакторном анализе. При многофакторном анализе ни один из исследованных факторов не был в значительной степени связан с недавними заражениями

вирусом Денге, хотя в двух местах проведения исследования, населённых в основном этническими китайцами, наблюдалась закономерность, предполагавшая возможность недавней вспышки. В большинстве случаев инфецирования не было не выявлено распознаваемого заболевания (вледствие бессимптомности либо неспецифичности симптомов), поскольку только 12,9% участников (31/240) смогли вспомнить, что в прошлом они уже болели лихорадкой денге.
Выводы: В выбранном для исследования преимущественно сельском сообществе обнаружился высокий уровень предшествующей подверженности лихорадке Денге. Выявление высокой доли незаегистрированных случаев, возможно, вследствие присутствия субклинических инфекций, подчёркивает необходимость расширенного наблюдения и методов контроля. Указанные результаты также влияют на измерение бремени болезни, на понимание динамики передачи заболевания, а также на предположительные последствия эффективности вакцины против вируса Денге и её разработки.

Translated from English version into Russian by Liudmila Tomanek, through

TRANSLATORS
WITHOUT BORDERS

Seroprevalencia del dengue entre adultos sanos en una comunidad rural del sur de Malasia: estudio piloto

Amreeta Dhanoa, Sharifah Syed Hassan, Nowrozy Kamar Jahan, Daniel D Reidpath, Quek Kia Fatt, Mohtar Pungut Ahmad, Cheong Yuet Meng, Lau Wee Ming, Prof. Anuar Zaini Zain, Prof. Maude Elvira Phipps, Prof. Iekhsan Othman, Aman Bin Rabu, Rowther Sirajudeen, Ahmad AbdulBasitz Ahmad Fatan, Dr. Faidzal Adlee Ghafar, Dr. Hamdan Bin Ahmad y Pascale Allotey

RESUMEN

Antecedentes: La frecuencia y la magnitud de las epidemias de dengue siguen aumentando exponencialmente en Malasia, con un cambio en el predominio de la franja de edad, que se inclina hacia la adultez, y con expansión en las zonas rurales. A pesar de esto, carecemos de información sobre la extensión de la transmisión del virus del dengue (DENV) en la comunidad rural. Hemos realizado este estudio piloto a nivel de la comunidad para establecer la seroprevalencia del DENV entre adultos sanos de un distrito rural del sur de Malasia e identificar sus factores determinantes.
Metodología: Para este estudio, llevado a cabo entre abril y mayo de 2015, se reclutó a un total de 277 participantes adultos entre las familias de tres localidades del subdistrito de Sungai Segamat, en el distrito de Segamat. Se analizó el suero sanguíneo para inmunoglobulina G (IgG) (Panbio® Dengue Indirect IgG ELISA/captura de alta carga viral) y para anticuerpos del tipo inmunoglobulina M (IgM) (Panbio®). Para confirmar el análisis, se realizó una prueba de reducción de placas por neutralización (PRNT) en muestras aleatorias de suero IgG positivo. Mediante entrevista, se recopilaron los historiales médicos y la historia previa de dengue; la información sociodemográfica se obtuvo de una base de datos preexistente.

Resultados: La seroprevalencia global de la infección por DENV ha sido de 86,6 % (240/277) (95% IC: 83–91%). Se han observado evidencias serológicas de infección reciente (IgM/captura de alta carga viral IgG) en un 11,2 % (31/277) de los participantes (IgG indirecto menos infecciones recientes). El ensayo de PRNT ha mostrado que los anticuerpos detectados eran específicos del DENV. El análisis multivariante ha mostrado que el grupo de mayor edad estaba asociado de forma significativa con pasadas infecciones por DENV. La seropositividad aumentaba con la edad: 48,5 % en el grupo de edad de <25 años hasta más de 85 % en el grupo de edad de >45 años ($P < 0.001$). No se han observado asociaciones con la ocupación, el lugar de estudios, el tipo de vivienda, la comorbilidad, el nivel educativo ni el estado civil; pero las dos últimas variables eran estadísticamente más significativas en el análisis univariable. Ninguno de los factores analizados se asoció de forma significativa con infecciones recientes por DENV en el análisis multivariable, aunque existía un patrón indiciario de un brote reciente en dos lugares analizados cuya población era predominantemente china. La mayoría de las infecciones no dio lugar a ninguna enfermedad reconocible (por ser asintomáticas o con síntomas no específicos); solo un 12,9 % de los participantes (31/240) refirieron haber tenido dengue en el pasado.

Conclusión: La comunidad sometida a análisis, predominantemente rural, tenía una exposición previa al dengue muy alta. El hallazgo de una gran proporción de casos sin notificar, posiblemente debido a infecciones subclínicas, subraya la necesidad de mejorar la vigilancia y los métodos de control. Este hallazgo tiene también implicaciones para la medición del impacto de la enfermedad, la comprensión de las dinámicas de transmisión y los efectos hipotéticos sobre la eficacia y la absorción de la vacuna contra el DENV.

Translated from English version into Spanish by Susana E. Cano Méndez, through

TRANSLATORS
WITHOUT BORDERS
